# Intrauterine growth restriction and its impact on intestinal morphophysiology throughout postnatal development in pigs

**DOI:** 10.1038/s41598-022-14683-z

**Published:** 2022-07-12

**Authors:** Thaís Garcia Santos, Saffir Dominique Fernandes, Stefany Bruna de Oliveira Araújo, Fernando Felicioni, Thaís de Mérici Domingues e Paula, André Lucas Caldeira-Brant, Soraia Viana Ferreira, Luciana de Paula Naves, Stefânia Priscilla de Souza, Paulo Henrique Reis Furtado Campos, Hélio Chiarini-Garcia, Ana Luísa Neves Alvarenga Dias, Fernanda Radicchi Campos Lobato de Almeida

**Affiliations:** 1 Laboratory of Structural Biology and Reproduction, Department of Morphology, Institute of Biological Sciences, Federal University of Minas Gerais, Antônio Carlos Avenue, 6627, Belo Horizonte, MG 31270-901 Brazil; 2 Department of Animal Science, Federal University of Lavras, University Campus, PO Box 3037, Lavras, MG 37200-000 Brazil; 3 Department of Animal Science, Federal University of Viçosa, Viçosa, MG 36570-900 Brazil; 4 Federal University of Uberlândia, BR 050 Km 78, Uberlândia, MG 38410-337 Brazil; 5 Department of Obstetrics, Gynecology, and Reproductive Sciences, Magee-Women’s Research Institute, University of Pittsburgh Medical Center, Pittsburgh, PA 15213 USA

**Keywords:** Cell biology, Physiology, Structural biology

## Abstract

Intrauterine growth restriction (IUGR) compromises fetal development, leading to low birth weight, and predisposes to gastrointestinal disorders. Pigs that suffered IUGR present poor postnatal development, resulting in great economic losses to the industry. The small intestine may be involved with impaired development, but studies investigating this issue are still limited. Thus, the present study aimed to investigate small intestine morphofunctional alterations in IUGR pigs throughout the production phases (birth to 150 days). IUGR pigs presented lower body weight from birth to the finishing phase (P < 0.05). Although histomorphometrical parameters were not affected during the pre-weaning period, their commitment was observed specifically in the duodenum of the IUGR group at older ages (P < 0.05). The most detrimental effects on the small intestine, such as deeper duodenum crypts’ depth, lower villus height:crypt depth ratio and absorptive area, increased apoptosis and lower proliferation of the duodenum epithelium were noticed at 70 days of age (P < 0.05). Additionally, IUGR pigs presented the lowest chymotrypsin and amylase activities at 70 and 150 days of age, respectively (P < 0.05). These findings may contribute to the elucidation of morphofunctional disorders of the small intestine in IUGR pigs throughout the different production phases, suggesting that poor postnatal development may be due to intestinal damage.

## Introduction

Genetic and nutritional strategies have been used to improve growth and reproductive performance in pigs. However, there are still some challenges that prevent animals to fully express their growth potential^1^. Among them, intrauterine growth restriction (IUGR) stands out as the main cause of low birth weight and postnatal impaired body growth, affecting up to 20% of piglets within a litter in modern hyperprolific sows^2,3^. It has been reported that IUGR has harmful impacts on neonatal survival, postnatal growth, nutrition utilization efficiency, health, and performance^2,4,5^. Thus, growth restriction in utero has major implications for animal agriculture^6^.

Animal physiology, metabolism and growth are directly dependent on the small intestine integrity and function, a key organ involved not only in the processes of digestion and absorption, but also in local and systemic immune responses^2,7^. There is increasing evidence that the uterine environment modulates intestinal development, so that insults at this stage can compromise its function, which might not be reversed throughout the postnatal life^8^. For instance, previous studies have reported villus atrophy and villus-crypt hyperplasia, delayed maturation of intestinal mucosa, decreased intestinal motility as well as decreased digestion and absorption of colostrum and milk in newborn and weaned IUGR pigs^9,10^. Although information on intestinal commitment has been previously reported, it involved mainly the weaning period^11^, with limited data on the other production phases.

Morphometrical and biochemical approaches are the most used techniques to evaluate small intestine structure and function, as they provide reliable results^12,13^. For example, intestinal maturity is extensively assessed by studying the size and number of villi, mucosal height, crypts depth, and enterocyte height^4,14,15^. In addition, as the activity of the brush border enzymes is considered an important indicator of maturation and digestive capacity in pigs^5,11^, its investigation in IUGR individuals becomes essential.

An adequate development of the gastrointestinal tract is critical to ensure the individual’s health. In this sense, an integrated and comprehensive understanding of growth, development and maturation of the intestine, and its dynamics throughout life, would contribute to the establishment of nutritional strategies to improve growth performance. Such knowledge will provide significant contribution to the increase of productivity and profitability for the swine industry^16^. Hence, the aim of the present study was to investigate the morphofunctional aspects of the small intestine in IUGR pigs throughout the postnatal development, from birth up to 150 days of age, and establish the critical production phase in which growth restriction in utero would have the most detrimental effects for subsequent growth performance.

## Methods

### Animals and experimental design

The experimental protocol was approved by the Animal Experimentation Ethics Committee (CEUA) from the Federal University of Minas Gerais (protocol nº 2016/342). All experiments were performed in accordance with the CEUA relevant guidelines and regulations. Reporting of all experimental procedures complied with recommendations in ARRIVE guidelines.

One hundred twenty pairs of male littermate piglets, born to 3rd to 6th parity sows, in litters of 15–22 total born and mean litter birthweight variation of 1.25–1.65 kg, were selected immediately after birth, before colostrum intake, and were divided into two birthweight categories: normal birthweight (NW), birthweight ranging from 1.6 to 1.9 kg (n = 120); and intrauterine growth restricted (IUGR), birthweight ranging from 0.7 to 1.0 kg (n = 120). All animals evaluated came from the same genetic lineage: crossbred F1 sows (Landrace × Large White) mated with Duroc—Agroceres PIC males. The NW–IUGR pair selected corresponded to the highest and the lowest birthweight males from each litter. The criteria used at selection were based on the concept of uterine crowding, according to previous studies^4,17^. To define the birthweight ranges, 1000 piglets of the same genotype were previously weighed, and the average and standard deviation calculated (μ = 1.3; ϭ = 0.3). Birthweight ranges were set as μ + ϭ to μ + 2ϭ for NW and μ − ϭ a μ − 2ϭ for IUGR^4,17–19^. All males were castrated between 5 to 7 days of age. Two sub-groups of 16 animals each (8 NW and 8 IUGR) were euthanized at either birth or 48 h after weaning (26 days of age), and two other sub-groups of 20 animals each (10 NW and 10 IUGR) were euthanized at either 70 or 150 days of age, to obtain biometrical data and tissue collection. Except for the newborn group, which was euthanized before colostrum intake, all animals were euthanized after an overnight fasting.

These ages were chosen based on the characterization of postnatal gastrointestinal maturation during the main stages of the production cycle: birth (reflects placental function); after weaning (transition from milk to solid food); 70 days (grower phase) and 150 days (finishing phase).

### Biometrical measures

Individual body weights were recorded at birth, 26 days, 70 days, and 150 days of age, without feed and water restriction. Immediately after euthanasia by electrical stunning and exsanguination, the small intestine was removed from the abdominal cavity, weighed and the length was recorded. Additionally, to confirm the occurrence of IUGR, brain and liver were weighed in the newborn subgroup to obtain the brain to liver weight ratio, as performed by Alvarenga et al.^4^.

### Sample collection and processing

Fragments from the duodenum and jejunum were collected from the cranial and duodenum-jejunal flexures, respectively, and submitted to different processing protocols. For histological analyzes, fragments (1–2 cm length) were washed in saline and the serous membranes were attached to the filter paper, fixed by immersion in 4% paraformaldehyde for 24 h, stored at 4 °C in 0.05 M phosphate buffer (pH 7.4) for 24 h and embedded in Paraplast (Sigma Aldrich, São Paulo, Brazil). Histological sections (5 µm thickness) were then deparaffinized in xylene and rehydrated with graded dilutions of ethanol. Slides were stained with hematoxylin and eosin (HE) and Periodic Acid Schiff (PAS) for morphological observations and goblet cell counting, respectively.

### Histomorphometrical evaluation

Histological sections were evaluated through a light microscope (Olympus BX51), and measurements performed using a ruler fitted in a 10× eyepiece, calibrated with a micrometer ruler. A total of 10 intact, well-oriented crypt-villus units from the duodenum and jejunum, were randomly selected for measurements of the following parameters, as previously described^4^: (a) mucosal height (MH): from the *muscularis mucosae* up to the apex of the villus; (b) villus height (VH), from the base up to the apex of the villus; (c) depth of the intestinal crypt (DC), from the *muscularis mucosa* to the base of the villus and (d) villus width (WV) and (e) crypt width (CW). Additionally, it was determined the ratio between villus height and intestinal crypt depth (VH/CD), villus surface area (S) and intestinal absorption area (AA), using the formulas described by Dong et al.^5^ and Kisielinski et al.^20^, as follows:

$$\mathrm{S}: \pi \frac{villus\, width}{2}\sqrt{{\left(\frac{villus\, width}{2}\right)}^{2}+{villus\, height}^{2}}$$

$$\mathrm{AA}: \frac{\left(villus\, width\cdot villus\, height\right)+{\left(\frac{villus\, width}{2}+ \frac{crypt\, widht}{2}\right)}^{2}- {\left(\frac{villus\, width}{2} \right)}^{2}}{\begin{array}{c}\left(\frac{villus\, width}{2}+ \frac{crypt\, width}{2}\right)\\ \end{array}}$$

Duodenum and jejunum histological sections were stained with Periodic Acid Schiff (PAS) to detect neutral mucins (stained in purple) present in goblet cells. The number of goblet cells was determined using the protocol standardized by Gomes et al.^21^. For each animal, ten random fields of the intestinal crypts were selected, and the images obtained using a light photomicroscope (BX-51 Olympus) connected to a Q-Color 3 digital camera (Olympus), at 400× magnification. All goblet cells were quantified in the crypt areas of each image using the Image-Pro Express software (Media Cybernetics, Rockville, MD, USA), and further equalized to squared millimeter (mm^2^).

### Expression of proliferation and apoptosis markers through immunofluorescence (IF)

To determine whether IUGR can have effects on proliferative activity and apoptosis of the duodenal epithelium, immunofluorescence assays for Ki67, a marker of cell proliferation, and Caspase-3, a marker of apoptosis, were performed. Five animals from the NW and IUGR groups were randomly selected. As the histomorphometrical parameters in the jejunum were similar in both experimental groups, proliferative and apoptotic assays were not performed. Briefly, the duodenum histological sections were dewaxed, rehydrated, and microwaved for 3 × 5 min in 0.1 M sodium citrate buffer (pH 6) for epitope antigen retrieval, cooled down to room temperature, and rinsed with phosphate-buffered saline pH7.4 (PBS). To reduce non-specific binding, sections were incubated with 3% bovine serum albumin (Sigma-Aldrich, São Paulo, Brazil) in Tris–HCl buffer solution (PBS, pH 7.3) for 90 min at 4 °C. All samples were incubated separately overnight at 4 °C with either primary antibody anti-Ki67 (IgG; 1:200; Thermo Fisher Scientific, São Paulo, Brazil) or anti-Caspase 3 (IgG; 1:600; Thermo Fisher Scientific, São Paulo, Brazil), respectively. Staining of all slides for each assay was carried out simultaneously in a single session. After washing, secondary antibody Alexa Fluor 555 (goat antirabbit, IgG, A-21422, Thermo Fisher Scientific, São Paulo, Brazil), diluted in 1:200 (antibody: PBS), was added to the sections and incubated for 90 min at 4 °C. Finally, nuclei were stained using DAPI (Sigma-Aldrich, São Paulo, Brazil), in the proportion 1:1000 (DAPI: PBS) and the slides were mounted with 50% glycerol (v/v in PBS). Negative controls were obtained by omitting the primary antibody. Human testes samples were used as the positive control for Ki-67, and canine breast tumor tissue was used for Caspase-3. Immunolocalization images were acquired using a Zeiss Apotome microscope (Carl Zeiss Microscopy, São Paulo, Brazil) equipped with filters suitable for detecting Alexa Fluor 555 signals.

To study cell proliferation, ten randomly selected field of intestinal gland (crypts) for each animal were photographed with a magnification of 200×. All positive cells in the epithelium of the intestinal gland were quantified using the Image-Pro Express software (Media Cybernetics, Rockville, USA) and the values expressed as number of immunostained cells per mm^2^ of intestinal gland.

On the other hand, Caspase-3 analysis was performed through the determination of fluorescence intensity. Photomicrographs were obtained at 200× magnification for each animal and 10 randomly selected intestinal villi delineated using the Image J software (NIH). Before analysis, the photomicrographs were converted to black and white images and the Image J software was equalized considering white as 100% (positive areas) and black as 0% of fluorescence intensity. Values were expressed as average % of fluorescence intensity.

### Determination of enzyme activity in the small intestine

To determine the enzymatic activities of lactase, amylase, lipase, chymotrypsin and trypsin, 2-cm samples of duodenum and jejunum were frozen in liquid nitrogen. For the preparation of the crude extract, 100 mg samples were homogenized in a Turrax type homogenizer, using 0.01 M phosphate buffered saline (NaCl 0.138 M; KCl—0.0027 M; pH 7.4) in a 1:10 ratio (weight:volume). After centrifugation at 15.000*g* for 15 min at 4 ºC, the supernatant was collected and stored at −20 ºC for further analysis.

The enzymes activities were measured in the duodenum and jejunum tissue extract samples. For all of them, the quantities of proteins in the enzyme extracts were determined according to Bradford^22^, using bovine serum albumin as the standard protein.

Lactase activity was determined using *O*-nitrophenyl *B*-d-galactopyranoside (ONPG, Sigma-Aldrich, São Paulo, Brazil) as an artificial substrate^23^. In the colorimetric assay, conducted at pH 7.4 and 37 °C, the ONPG was cleaved by lactase releasing ortho-nitrophenol that was measured at 420 nm. The chymotrypsin activity was measured according to the Hummel method^24^ based in the hydrolysis rate of the substrate *N*-Benzoyl-l-tyrosine ethyl ester (BTEE, Sigma-Aldrich, São Paulo, Brazil). The trypsin activity was determined using N*α* Benzoyl-dl-arginine 4-nitroanilide hydrochloride (BAPNA, Sigma-Aldrich, São Paulo, Brazil) as a substrate according to the method of Erlanger et al.^25^. Finally, lipase activity was determined according to the protocol described in the Lipase assay kit (Analisa Gold, Belo Horizonte, Brazil) based on an improved dimercaptopropanol tributyrate (BALB) method, in which SH groups were formed from the lipase cleavage of BALB reaction with 5,5'-dithiobis (2-nitrobenzoic acid) (DTNB) to originate a yellow-colored product. The color intensity, measured at 412 nm, was proportional to the enzyme activity in the sample. For the determination of amylase activity, the Amylase assay kit was used (Analisa Gold, Belo Horizonte, Brazil) based on the modified Caraway method: the amylase contained in the sample hydrolyzed the starch by releasing sugar and dextrin molecules. After the addition of the iodine solution, the blue color was formed by complexation with the non-hydrolyzed starch. Thus, the amylase activity was inversely proportional to the intensity of the blue color formed and was calculated in comparison to the substrate control.

The specific activity of all enzymes was expressed in U/mg protein, being U defined as the amount of enzyme that hydrolyzes 1 mol of substrate per minute of reaction. All samples were analyzed in duplicate and the absorbance values were measured using the Power Wave™ XS Microplate Scaring Spectrophotometer (Bio-Tek Instruments, Potton, United Kingdom).

### Statistical analyses

All variables were tested for normality prior to analysis, using the univariate procedure of the Statistical Analysis System^26^. Data were analyzed as a randomized complete block design, with littermate pairs used as block, and pig was the experimental unit. Data were submitted to analysis of variance (ANOVA), performed by the General Linear procedure of SAS and means were compared by the Student-T test, with P < 0.05 considered significant. Important associations between performance and small intestine parameters were evaluated across birthweight groups by correlation analysis (INSIGHT procedure of SAS). In tables and figures, data are reported as least square means and the pooled SEM.

### Ethical approval

The experimental protocol was approved by the Animal Experimentation Ethics Committee from the Federal University of Minas Gerais (protocol no. 2016/342).

## Results

### Growth performance and biometrical parameters

**Figure 1:**
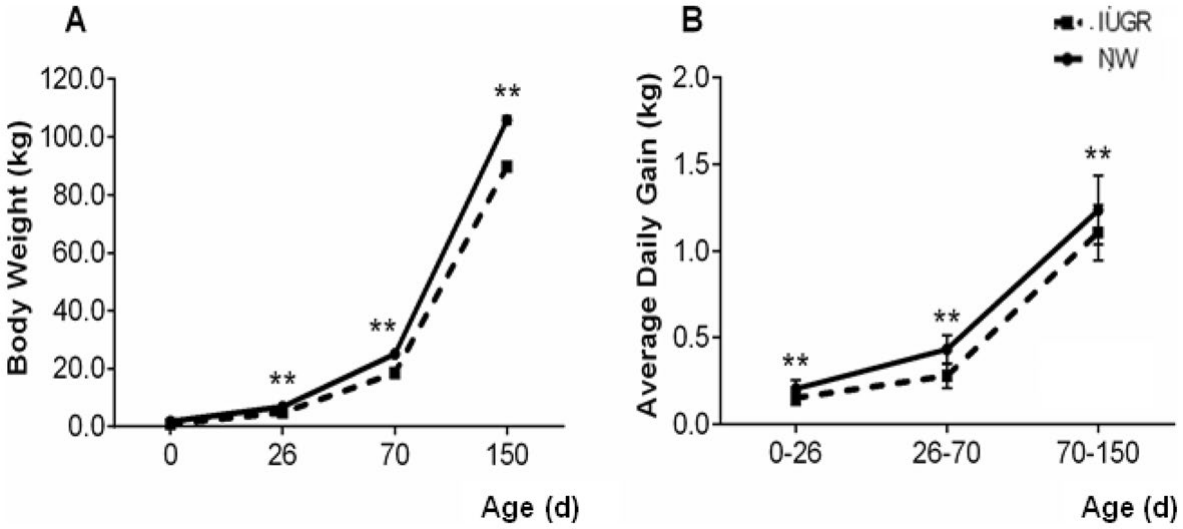
Growth curve (**A**) and average daily gain (**B**) in growth restricted (IUGR) and normal (NW) birth weight male littermate pigs from birth (0) to 150 days of age. IUGR pigs grew slower and gained less body weight throughout the production phases. Double asterisk: means differ statistically between groups (P < 0.001). Error bars represent the standard errors of the means.

Body weight and average daily gain from birth to 150 days of age are presented in Fig. 1. Intrauterine growth-restricted piglets showed lower body weights during all stages of development compared to their NW littermates (P < 0.001). In IUGR piglets, the average daily weight gain for the first phase (0–26 days) was 0.153 g, for the second phase (27–70 days) 0.280 g and for the third phase (71–150 days), 1.106 g. These values were lower than the NW animals, whose gains were, respectively, 0.206 g, 0.433 g and 1.235 g (P < 0.001). In addition to poor performance, IUGR pigs also showed higher mortality rate throughout the production phases (22 animals in the IUGR group vs 10 animals in the NW group).

Furthermore, newborn IUGR piglets presented lower liver and small intestine weights, and shorter small intestine length, which persisted up to 70 days of age (P < 0.05). However, brain:liver weight ratio was higher in the IUGR experimental group, providing strong evidence of intrauterine growth restriction in this experimental group (P < 0.05). Although the small intestine weight and size were affected by birth weight up to 70 days of age, its relative weight and size were similar in both experimental groups at all ages evaluated except at 70 days, when those parameters were higher in IUGR animals (P < 0.05; Table 1).

**Table 1:** Body weight and intestinal biometrical parameters from normal (NW) and intrauterine growth restricted (IUGR) male littermate pigs throughout the production phases.

Parameters	Birth			26 days			70 days			150 dias		
	NW (n = 8)	IUGR (n = 8)	*P*-value	NW (n = 10)	IUGR (n = 10)	*P*-value	NW (n = 10)	IUGR (n = 10)	*P*-value	NW (n = 10)	IUGR (n = 10)	*P*-value
Body weight (kg)	1.62 ± 0.01^a^	0.87 ± 0.01^b^	0.005	6.6 ± 0.10^a^	4.8 ± 0.10^b^	0.001	25.0 ± 0.40^a^	18.5 ± 0.40^b^	0.001	106.0 ± 1.30^a^	90.0 ± 1.30^b^	0.032
SI weight (kg)	0.060 ± 0.005^a^	0.030 ± 0.005^b^	0.002	0.2 ± 0.2^a^	0.1 ± 0.2^b^	0.034	1.6 ± 0.7^a^	1.2 ± 0.7^b^	0.003	3.1 ± 0.2^a^	3.0 ± 0.2^a^	0.074
SI length (m)	4.0 ± 0.1^a^	2.6 ± 0.1^b^	0.0001	7.2 ± 0.2^a^	6.7 ± 0.2^b^	0.035	15.0 ± 0.4^a^	13.6 ± 0.4^b^	0.012	18.5 ± 0.6^a^	18.0 ± 0.5^a^	0.621
Relative SI weight	0.03 ± 0.002^a^	0.03 ± 0.006^a^	0.678	0.04 ± 0.004^a^	0.04 ± 0.002^a^	0.830	0.05 ± 0.002^a^	0.07 ± 0.007^b^	0.039	0.02 ± 0.001^a^	0.03 ± 0.002^a^	0.074
Relative SI length	2.52 ± 0.12^a^	3.30 ± 0.30^a^	0.070	1.44 ± 0.15^a^	1.64 ± 0.18^a^	0.975	0.55 ± 0.02^a^	0.80 ± 0.09^b^	0.014	0.16 ± 0.008^a^	0.19 ± 0.014^a^	0.498
Brain weight (g)	27.5 ± 0.5^a^	24.4 ± 0.5^b^	0.003	–	–		–	–		–	–	
Liver weight (g)	52.5 ± 4.3^a^	20.5 ± 4.3^b^	0.001	146.5 ± 9.1^a^	101.8 ± 9.1^b^	0.006	917.0 ^a^ ± 39.2^b^	672.0^a^ ± 39.2^b^	0.0003	1839.0 ± 128^a^	1864.0 ± 128^a^	0.894
Brain:liver	0.55 ± 0.07^a^	1.24 ± 0.07^b^	0.0003	–	–		–	–		–	–	

Relative SI weight: small intestine (kg)/body weight (kg). Relative SI length: small intestine (m)/body weight (kg).

*SI* small intestine.

^ab^Within the same age, least square means with different superscripts within a row differ (P < 0.05).

### Small intestine histomorphometry and kinetics

The small intestine histomorphometrical data are summarized in Table 2. From birth to weaning, the duodenum was not structurally affected by IUGR. However, the negative effects of IUGR became evident at the grower (70 days) and finishing (150 days) stages, with a significant reduction in villus height, absorptive area, and villus surface area (P < 0.01). IUGR effects on the intestinal mucosa were more severe at 70 days since an increase in crypt depth and a decrease in the villus / crypt ratio were observed (P < 0.05).

**Table 2:** Small intestine histomorphometrical parameters from normal (NW) and intrauterine growth restricted (IUGR) male littermate pigs throughout the production phases.

Parameters	Birth			26 days			70 days			150 days		
	NW (n = 8)	IUGR (n = 8)	*P*-value	NW (n = 10)	IUGR (n = 10)	*P*-value	NW (n = 10)	IUGR (n = 10)	*P*-value	NW (n = 10)	IUGR (n = 10)	*P*-value
**Duodenum**												
Villus height (µm)	416 ± 12^a^	398 ± 10^a^	0.342	334 ± 47^a^	323 ± 47^a^	0.875	313 ± 23^a^	193 ± 23^b^	0.007	395 ± 39^a^	246 ± 39^b^	0.036
Crypt depth (µm)	125 ± 7^a^	111 ± 7^a^	0.217	324 ± 15^a^	288 ± 15^a^	0.140	441 ± 21^a^	526 ± 15^b^	0.023	567 ± 70^a^	555 ± 70^a^	0.909
Mucosal height (µm)	557 ± 53^a^	571 ± 29^a^	0.348	536 ± 103^a^	486 ± 115^a^	0.476	833 ± 37^a^	785 ± 37^a^	0.433	1054 ± 208^a^	1236 ± 205^a^	0.355
Villus/crypt ratio	3.4 ± 0.3^a^	4.0 ± 0.3^a^	0.192	1.0 ± 0.1^a^	0.9 ± 0.1^a^	0.760	0.7 ± 0.1^a^	0.4 ± 0.1^b^	0.002	0.7 ± 0.1^a^	0.5 ± 0.1^a^	0.110
V.S.A (mm^2^)	0.61 ± 0.07^a^	0.47 ± 0.07^a^	0.194	0.13 ± 0.02^a^	0.12 ± 0.0^a^	0.903	0.015 ± 0.02^a^	0.07 ± 0.02^b^	0.012	0.25 ± 0.02^a^	0.13 ± 0.02^b^	0.006
Absorptive area	0.030 ± 0.004^a^	0.024 ± 0.004^a^	0.620	0.06 ± 0.01^a^	0.05 ± 0.01^a^	0.350	0.09 ± 0.01^a^	0.06 ± 0.01^b^	0.034	0.080 ± 0.004^a^	0.050 ± 0.004^b^	0.002
G.C/crypt (mm^2^)	3415 ± 350^a^	3306 ± 477^a^	0.714	3222 ± 169^a^	2862 ± 252^a^	0.683	2748 ± 257^a^	3209 ± 481^a^	0.522	4238 ± 254 ^a^	4145 ± 256 ^a^	0.785
**Jejunum**												
Villus height (µm)	760 ± 52^a^	577 ± 48^a^	0.455	249 ± 20^a^	225 ± 9^a^	0.914	372 ± 21^a^	337 ± 19^a^	0.882	395 ± 39^a^	246 ± 39^a^	0.587
Crypt depth (µm)	100 ± 2^a^	73 ± 3^a^	0.221	201 ± 21^a^	184 ± 13^a^	0.467	328 ± 31^a^	326 ± 18^a^	0.667	567 ± 70^a^	555 ± 70^a^	0.9888
Mucosal height (µm)	328 ± 102^a^	665 ± 44^a^	0.144	560 ± 31^a^	540 ± 36^a^	0.835	755 ± 32^a^	655 ± 32^a^	0.469	1054 ± 208^a^	1236 ± 205^a^	0.588
Villus/crypt ratio	8.7 ± 0.7^a^	7.8 ± 0.4^a^	0.423	1.3 ± 0.1^a^	1.1 ± 0.0^a^	0.766	1.2 ± 0.1^a^	1.0 ± 0.1^a^	0.651	0.7 ± 0.1^a^	0.5 ± 0.1^a^	0.833
V.S.A (mm^2^)	0.07 ± 0.10^a^	0.06 ± 0.04^a^	0.645	0.04 ± 0.01^a^	0.03 ± 0.01^a^	0.844	0.06 ± 0.01^a^	0.06 ± 0.01^a^	0.977	0.25 ± 0.02^a^	0.13 ± 0.02^a^	0.566
Absorptive area	0.030 ± 0.001^a^	0.030 ± 0.001^a^	0.894	0.040 ± 0.004^a^	0.030 ± 0.001^a^	0.721	0.010 ± 0.002^a^	0.010 ± 0.002^a^	0.945	0.080 ± 0.004^a^	0.050 ± 0.004^a^	0.760
G.C/crypt (mm^2^)	2455 ± 310^a^	3104 ± 277^a^	0.521	3215 ± 157^a^	2562 ± 252^a^	0.682	2345 ± 257^a^	3217 ± 281^a^	0.562	3615 ± 244^a^	3288 ± 235^a^	0.185

Villus/crypt ratio: villus height/crypt depth.

*V.S.A* villus surface area. *G.C/crypt* goblet cell number per crypt.

^ab^Within the same age, least square means with different superscripts within a row differ (P < 0.05).

**Figure 2:**
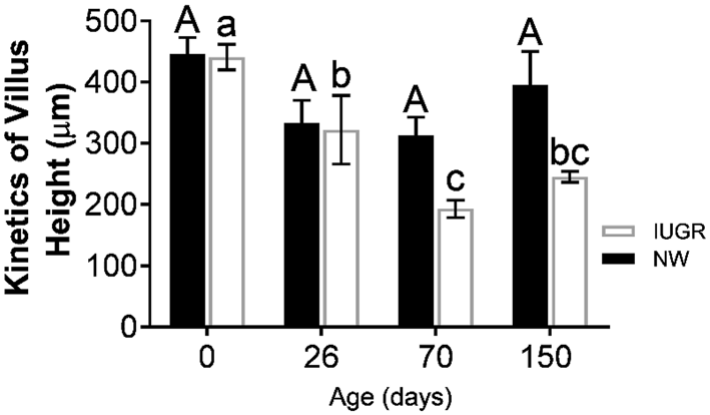
Villus height of the duodenum mucosa throughout postnatal growth (kinetics) in growth restricted (IUGR) and normal (NW) birth weight male littermate pigs. Villus height decreased sharply from birth to 70 days of age in IUGR pigs (black square), reaching its lowest height at this age. On the other hand, villus height remained relatively stable in the NW littermates in the same period. Superscript a, b: means differ statistically between groups (P < 0.05). Error bars represent the standard errors of the means.

Figure 2 shows aspects of the duodenal mucosa developmental kinetics (from birth to 150 days of age). Interestingly, in the NW group, villus height was relatively stable from birth to finishing. On the other hand, in IUGR pigs, duodenal villi height decreased after weaning and persisted low until 150 days of age, with the lowest height observed at 70 days.

Moreover, histomorphometrical parameters in the jejunum were similar in both experimental groups at all ages evaluated, suggesting that the duodenum is the segment of the small intestine that is more sensitive to IUGR effects on the mucosa.

### Evaluation of cellular proliferation and apoptosis in the duodenum epithelium

**Figure 3:**
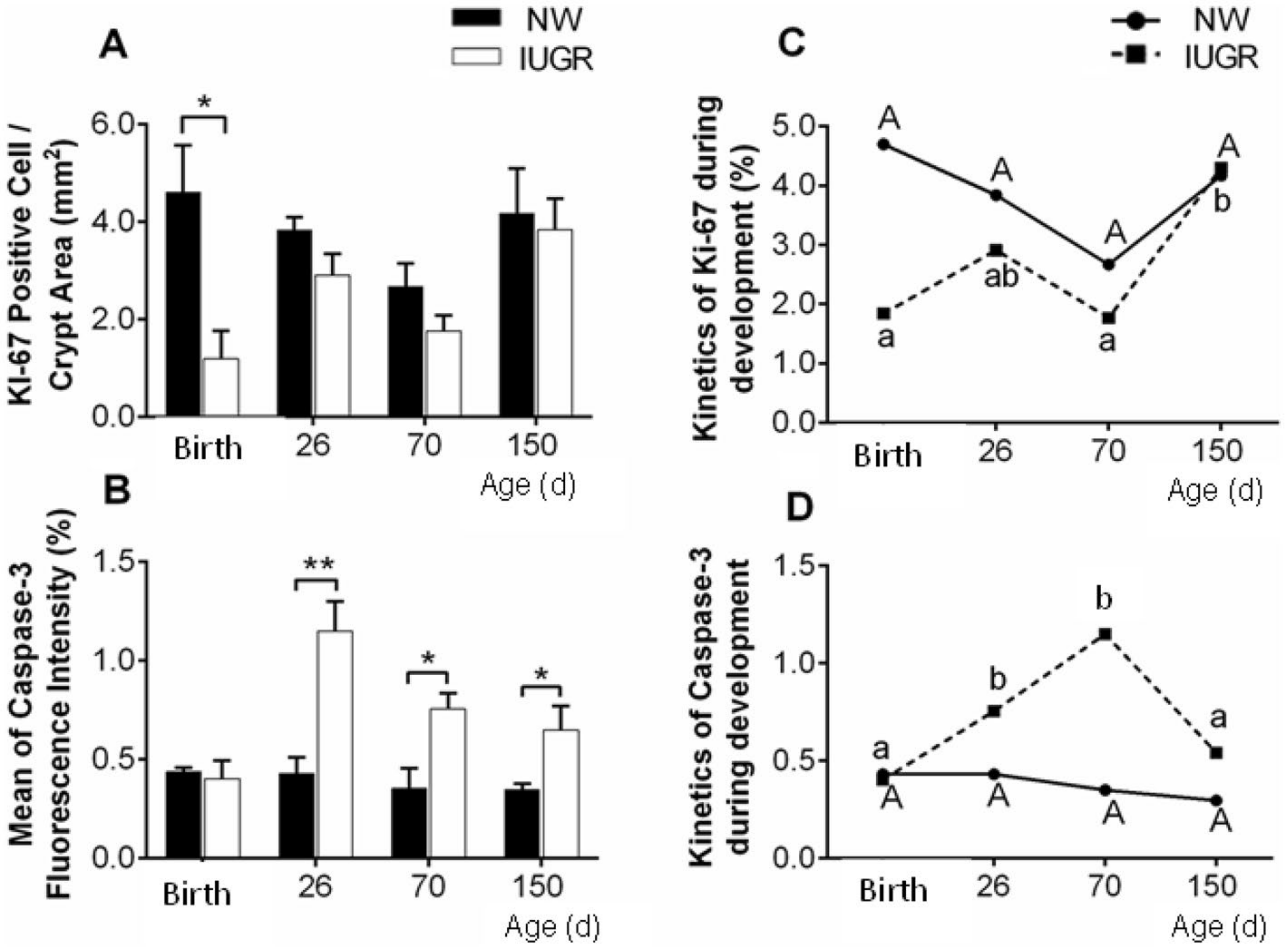
Characterization of Ki-67 and Caspase-3 expressions in the duodenum of intrauterine growth restricted (IUGR) and normal (NW) birth weight male littermate pigs. (**A**) Number of Ki-67 positive cells per area of epithelium of intestinal glands (density). Cellular proliferation was higher in NW piglets at birth, but remained similar between the experimental groups throughout the production phases. (**B**) Mean percentage of Caspase-3 fluorescence cells in the duodenal villi. IUGR pigs presented higher apoptosis after weaning, which remained higher until 150 days of age. (**C**) The pattern of cell proliferation overtime (from birth to 150 days of age) in NW pigs remained relatively constant throughout postnatal development. On the other hand, in IUGR pigs, cell proliferation significantly increased from 70 to 150 days of age (**D**). Regarding the pattern of apoptosis overtime, mean percentage of Caspase-3 fluorescence in the duodenal villi remained constant in the NW animals, but it increased drastically until 70 days in the IUGR pigs. Superscript a, b: LS means differ statistically between groups (**P < 0.01 and *P < 0.05). Error bars represent standard errors of the means.

The results of cellular proliferation and apoptosis are shown in Fig. 3. Growth restriction in utero negatively affected cellular proliferation of the duodenum epithelium, which was lower in IUGR piglets at birth (Fig. 3A; P < 0.05). However, this parameter was similar throughout the production phases (from weaning to 150 days of age).

Regarding apoptosis of the duodenum epithelium, a different pattern of expression was observed. Although it was similar between the experimental groups at birth, Caspase-3 expression increased overtime, reaching the highest value at 26 days of age (Fig. 3B; P < 0.01) and continued to be higher at 70 and 150 days of age (Fig. 3B; P < 0.05).

When comparing the KI-67 expression between ages in the same experimental group, the NW group showed a homogeneous pattern from birth to 150 days of age, but in the IUGR group, there was a marked increase between 70 and 150 days of age (Fig. 3C; P < 0.05). On the other hand, when comparing the Caspase-3 expression between ages in the same group, a sharp increase was observed when the piglets were 26 and 70 days old, however at 150 days the expression dropped and reached a similar expression presented at birth (Figs. 3D; P < 0.05).

### Body weight and intestinal parameters correlations

The correlations between body weight and duodenum histomorphometrical parameters are presented in Table 3. Intestinal weight and length, as well as villus height, villus height:crypth depth ration, absorptive area and Ki-67 expression were positively correlated with body weight at all ages. Conversely, Caspase-3 expression was negatively correlated to body weight, as it was higher when body weight was lower (P < 0.05). Interestingly, these associations seemed more evident particularly at 70 days of age, reflecting late IUGR effects on the small intestine.

**Table 3:** Correlations between small intestine morphofunctional parameters and body weight at birth, 26 days, 70 days, and 150 days of age.

Parameters	Birthweight		Body weight at 26 days		Body weight at 70 days		Body weight at 150 days	
	r	*P*-value	r	*P*-value	r	*P* -value	r	*P-*value
SI weight	0.86	0.001	0.83	0.011	0.69	0.027	0.49	0.214
SI length	0.87	0.0009	0.65	0.083	0.89	0.0005	0.90	0.002
Villus height	0.18	0.638	0.26	0.530	0.68	0.029	0.60	0.115
Crypt depth	0.11	0.756	0.40	0.330	−0.64	0.044	–0.22	0.600
Villus/crypt ratio	−0.23	0.506	0.35	0.440	0.76	0.011	0.56	0.147
Absorptive area	−0.08	0.820	0.50	0.203	0.60	0.066	0.67	0.096
Villus surface area	0.30	0.399	0.24	0.570	0.67	0.035	0.72	0.042
Ki-67	0.69	0.026	0.72	0.042	0.63	0.053	0.19	0.660
Caspase-3	0.24	0.505	−0.66	0.076	−0.80	0.006	−0.71	0.047

A positive correlation was observed between body weight and cell proliferation at all ages evaluated. On the other hand, for the apoptosis marker, a negative correlation with body weight from weaning to 150 days was observed, indicating that, in this period, the lower the body weight, the higher the apoptosis index in the duodenal epithelium.

### Digestive enzymes activity assays

Table 4 presents the results of specific enzymes activity in the duodenum and jejunum. In the duodenum, a decrease in amylase activity was observed in IUGR pigs at 150 days of age (P < 0.05), whereas the activities of lactase, lipase, trypsin and chymotrypsin were not affected by birthweight at the other ages evaluated. In the jejunum, there was a decrease in the activity of chymotrypsin in the IUGR group at 70 days of age (P < 0.05), while the other enzymes (lactase, lipase, amylase and trypsin) activities were similar between both experimental groups. No changes in enzymes activities were observed at birth or at weaning (P > 0.05).

**Table 4:** Enzymatic activity in the small intestine enzymes from normal (NW) and intrauterine growth restricted (IUGR) male littermate pigs throughout the production phases.

Parameters	Birth			26 days			70 days			150 dias		
	NW (n = 8)	IUGR (n = 8)	*P*-value	NW (n = 10)	IUGR (n = 10)	*P*-value	NW (n = 10)	IUGR (n = 10)	*P*-value	NW (n = 10)	IUGR (n = 10)	*P*-value
**Duodenum**												
Lactase (U/mg)	383 ± 93^a^	417 ± 93^a^	0.752	162 ± 35^a^	132 ± 35^a^	0.355	92 ± 13^a^	73 ± 12^a^	0.722	71 ± 11^a^	84 ± 11^a^	0.557
Amylase (U/mg)	1.00 ± 0.40^a^	1.55 ± 0.30^a^	0.466	1.65 ± 0.40^a^	0.39 ± 0.50^a^	0.267	1.80 ± 0.20^a^	1.55 ± 0.20^a^	0.681	1.31 ± 0.10^a^	0.85 ± 0.10^b^	0.021
Lipase (U/mg)	0.12 ± 0.10^a^	0.19 ± 0.10^a^	0.233	0.08 ± 0.10^a^	0.05 ± 0.10^a^	0.433	0.04 ± 0.10^a^	0.06 ± 0.10^a^	0.455	0.03 ± 0.10^a^	0.02 ± 0.10^a^	0.663
Chymotrypsin (U/mg)	579 ± 150^a^	201 ± 28^a^	0.162	237 ± 53^a^	267 ± 59^a^	0.899	330 ± 80^a^	376 ± 76^a^	0.223	731 ± 104^a^	532 ± 127^a^	0.768
Trypsin (U/mg)	779 ± 165^a^	400 ± 113^a^	0.447	406 ± 91^a^	242 ± 105^a^	0.655	382 ± 103^a^	635 ± 98^a^	0.552	295 ± 73^a^	115 ± 79^a^	0.388
**Jejunum**												
Lactase (U/mg)	287 ± 97^a^	407 ± 97^a^	0.322	227 ± 75^a^	275 ± 75^a^	0.786	234 ± 57^a^	216 ± 40^a^	0.442	88 ± 23^a^	128 ± 21^a^	0.766
Amylase (U/mg)	1.0 ± 0.30^a^	1.2 ± 0.40^a^	0.123	1.07 ± 0.40^a^	1.28 ± 0.30^a^	0.557	0.97 ± 0.20^a^	0.58 ± 0.20^a^	0.664	0.61 ± 0.10^a^	0.56 ± 0.10^a^	0.377
Lipase (U/mg)	0.04 ± 0.10^a^	0.04 ± 0.10^a^	0.992	0.04 ± 0.10^a^	0.05 ± 0.10^a^	0.332	0.08 ± 0.10^a^	0.05 ± 0.10^a^	0.756	0.04 ± 0.10^a^	0.07 ± 0.10^a^	0.574
Chymotrypsin (U/mg)	461 ± 77^a^	387 ± 62^a^	0.688	402 ± 301^a^	663 ± 301^a^	0.225	367 ± 55^a^	190 ± 62^b^	0.036	340 ± 75^a^	402 ± 86^a^	0.277
Trypsin (U/mg)	555 ± 79^a^	460 ± 62^a^	0.955	494 ± 116^a^	354 ± 150^a^	0.446	474 ± 99^a^	372 ± 99^a^	0.142	272 ± 48^a^	199 ± 64^a^	0.421

^ab^Within the same age, least square means with different superscripts within a row differ (P < 0.05).

## Discussion

There is evidence that intestinal integrity is a key factor for intestinal health in pigs^7^. In the present study, we focused on the changes in the small intestine mucosa and enzymes activities in IUGR pigs throughout the production phases (from birth to the finishing period). In general, studies performed to investigate the functionality of the small intestine are limited to the weaning phase^11^. However, the present study demonstrated, for the first time, intestinal morphofunctional alterations in intrauterine growth restricted pigs from birth to 150 days of age, showing evidence that the most detrimental effects on the small intestine occur at the grower period (70 days of age).

We observed that the weight and length of the small intestine in IUGR animals remained lower until 70 days, which may be due to a high rate of apoptosis in the intestinal epithelium. This finding may indicate lower nutrient utilization and provide the physiological basis for poor postnatal body growth in these animals^27^. Interestingly, intestinal length relative to body weight was greater in IUGR pigs but its consequences remain unclear, as the small intestine weight was lower. Nevertheless, other authors have observed that in IUGR pigs, although presenting a relatively longer SI, it is also thinner, and such intestinal vulnerability may explain the higher frequency and severity of intestinal problems in these animals^14,28^.

Our data showed that IUGR did not affect the duodenal morphology during the preweaning period. However, villus height, surface area, absorption area and villus / crypt ratio decreased along the postweaning period. Such commitment was evident when villus height was compared overtime within the same experimental group (kinetics), as a significant drop was observed, in particular, at 70 days of age. The greater crypt depth in the IUGR group at 70 days suggests that even though there is proliferative activity of stem cells , it does not compensate for cell losses, since the villi remained shorter. This result is relevant, since intestinal commitment during that specific production phase may compromise the proper digestion of feed ingredients, thus altering nutrient availability^1,3^.

The immune and digestive systems of the newly weaned piglets are still immature. Thus, these animals are naturally more susceptible to enteric challenges, leading to less body weight gain, which might be compensated over time^5,29^. In this study, commitment of the intestinal integrity in IUGR pigs during the growing phase provides evidence of deficient recovery of the intestinal epithelium. Given this scenario, it is worth mentioning that both experimental groups shared the same genetics, lactation conditions and received the same diets.

To understand the dynamics of intestinal epithelium cellular turn-over, expressions of proliferation and apoptosis markers were evaluated. Caspase-3 is a marker of cell apoptosis and plays an important role in the exchange of intestinal epithelial cells^30^. Higher rates of apoptotic cells in the duodenum villus were observed in IUGR piglets from weaning to the finishing phase. This finding suggests that the greater loss of enterocytes may cause shortening of the villi^31^, which might be a factor involved in the delay of the intestinal epithelium maturation. Furthermore, higher apoptosis may affect the integrity of the intestinal mucosa, such as permeability of the intestinal barrier^10^, which might be partly explained by a higher incidence of diarrhea in low birthweight piglets at commercial farms.

Our data showed a significant decrease in the expression of Ki-67, a cell proliferation marker, in the intestinal glands of IUGR newborns. Since proliferative cells of the intestinal epithelium include stem / progenitor cells, stem cells may have reduced regenerative capacity in the immature intestine of IUGR individuals^32^. This limited ability of damage repair can accentuate intestinal injury, especially when the intestine is submitted to insults during feeding changes along the postweaning period.

When comparing the expressions of Ki-67 and Caspase-3 overtime within the same experimental group, in the NW group they were similar throughout postnatal life, whereas in IUGR pigs there were variations for both markers over time. For example, the expression of Ki-67 was greater at 150 days, whereas, at that same age, the expression of Caspase-3 decreased. These events may reflect a compensatory mechanism of mucosal recovery, after the renewal disorders observed at 70 days of age.

Although morphological changes have been observed, impairments in enzyme activities were mild. We demonstrated that, in the duodenum and jejunum of IUGR piglets, lactase activity was not significantly affected. In addition to lactase, lipase and trypsin were not affected by growth restriction in utero at any of the production phases, but the jejunum chymotrypsin decreased by 48% at 70 days of age and duodenal amylase secretion was 35% lower at 150 days. Chymotrypsin and trypsin catalyze the hydrolysis of the peptide bonds of dietary protein, while amylase degrades carbohydrates in the mammalian intestine^14^. In this sense, the lower enzymatic activity of both enzymes in the grower and finishing phases can be a limiting factor for efficient digestion of proteins and carbohydrates, essential nutrients for optimal muscle growth.

The post weaning is a crucial period of intense duodenal disorders, particularly the transition from the nursery to the grower periods. Thus, this transition deserves special attention, and management and nutritional practices should be implemented to minimize those deleterious effects. Taken together, our findings suggest that permanent low growth performance of IUGR pigs may be a consequence of duodenal damage, not at weaning, but at the grower period.

## Conclusion

The results obtained in the present study provide evidence that growth restriction during prenatal life contributes in various ways to the impairment of postnatal development until the finishing phase, expressed by reduction of villi height, decreased absorption area and reduced activity of important enzymes for the digestion of proteins and carbohydrates. Our findings may support the concept of intrauterine programming on the risk of gastrointestinal disorders, with the end of the nursery period (70 days) representing the crucial phase of intense duodenal disturbances. Such findings may contribute to the elucidation of morphological and functional disorders in IUGR pigs at different production phases, shedding light to the implementation of specific nutritional management to improve productivity and increase profitability for the swine industry.

## Data Availability

All data supporting our findings are included in the manuscript.
